# Whole genome sequencing in support of wellness and health maintenance

**DOI:** 10.1186/gm462

**Published:** 2013-06-27

**Authors:** Chirag J Patel, Ambily Sivadas, Rubina Tabassum, Thanawadee Preeprem, Jing Zhao, Dalia Arafat, Rong Chen, Alexander A Morgan, Gregory S Martin, Kenneth L Brigham, Atul J Butte, Greg Gibson

**Affiliations:** 1 Division of Systems Medicine, Department of Pediatrics, Stanford University School of Medicine, 251 Campus Drive, Palo Alto, CA 94304, USA; 2 Lucille Packard Children's Hospital, 725 Welch Rd, Palo Alto, CA 94304, USA; 3 School of Biology, Georgia Institute of Technology, 310 Ferst Drive, Atlanta GA 30332, USA; 4 Personalis, Inc., 1350 Willow Rd Suite 202, Menlo Park, CA 94025, USA; 5 Center for Health Discovery and Well Being, and School of Medicine, Emory University Midtown Hospital, 550 Peachtree St, Atlanta GA 30308, USA

**Keywords:** genetic prediction, risk assessment, preventive medicine, clinical profiling

## Abstract

**Background:**

Whole genome sequencing is poised to revolutionize personalized medicine, providing the capacity to classify individuals into risk categories for a wide range of diseases. Here we begin to explore how whole genome sequencing (WGS) might be incorporated alongside traditional clinical evaluation as a part of preventive medicine. The present study illustrates novel approaches for integrating genotypic and clinical information for assessment of generalized health risks and to assist individuals in the promotion of wellness and maintenance of good health.

**Methods:**

Whole genome sequences and longitudinal clinical profiles are described for eight middle-aged Caucasian participants (four men and four women) from the Center for Health Discovery and Well Being (CHDWB) at Emory University in Atlanta. We report multivariate genotypic risk assessments derived from common variants reported by genome-wide association studies (GWAS), as well as clinical measures in the domains of immune, metabolic, cardiovascular, musculoskeletal, respiratory, and mental health.

**Results:**

Polygenic risk is assessed for each participant for over 100 diseases and reported relative to baseline population prevalence. Two approaches for combining clinical and genetic profiles for the purposes of health assessment are then presented. First we propose conditioning individual disease risk assessments on observed clinical status for type 2 diabetes, coronary artery disease, hypertriglyceridemia and hypertension, and obesity. An approximate 2:1 ratio of concordance between genetic prediction and observed sub-clinical disease is observed. Subsequently, we show how more holistic combination of genetic, clinical and family history data can be achieved by visualizing risk in eight sub-classes of disease. Having identified where their profiles are broadly concordant or discordant, an individual can focus on individual clinical results or genotypes as they develop personalized health action plans in consultation with a health partner or coach.

**Conclusion:**

The CHDWB will facilitate longitudinal evaluation of wellness-focused medical care based on comprehensive self-knowledge of medical risks.

## Background

Whole genome sequencing (WGS) and exome sequencing are rapidly being incorporated as routine components of diagnosis and explanation of rare disorders, and the trend is moving toward utilization of these for risk assessment for common diseases as well [1,2]. Each month, novel mutations (either *de novo *or transmitted) that are causal for conditions such as autism, primary immunodeficiency, and craniofacial abnormalities are reported [3-8]. In parallel, widespread adoption of genome-wide association studies (GWAS) have identified thousands of loci that contribute to multifactorial diseases as diverse as diabetes, asthma, and depression [9,10]. Because environmental factors make a substantial contribution to these latter conditions, 'prediction' is too strong a claim for genomic medicine [11,12], but risk stratification is certainly feasible [13]. Here we explore how WGS might be incorporated alongside traditional clinical evaluation as part of preventive medical care and the maintenance of good health. The term 'whole genome sequencing' does not imply that the entire genome of each individual is sequenced, but rather should be taken to mean that total genomic DNA was sequenced to high depth, in contrast to targeted or whole exome sequencing.

We have previously shown in multiple settings how GWAS results can be used in combination with personal WGS to evaluate individual risk. The probability of developing each of approximately 100 diseases was estimated for individuals by integrating allelic risk effects for multiple well-validated common variants with population-specific pre-test baseline lifetime risk of disease estimates. We first showed that the pseudo-individual represented by the Human Reference Genome (hg19) would have an increased risk for type 1 diabetes (T1D) [14]. We then reported on three Caucasian individuals. The first had known familial risk of heart disease, which was borne out by his genomic profile of both common variant and rare deleterious variant-associated risk for coronary artery disease (CAD) [15]. The second was found to have an unexpected predisposition to type 2 diabetes (T2D), which revealed itself in an extended period of hyperglycemia following a respiratory viral infection [16], and parallel longitudinal transcriptomic, proteomic, and immune profiling supported the inference of a change in health status. For the third case, the immunological and pharmacological risk assessment was advanced by incorporating new methodologies for analysis of family quartets [17]. Most recently, we also evaluated the WGS of a South Asian woman from Kerala [18], and showed how genetic risk distribution for a number of diseases varies in different populations [19].

Although the focus of most genomic medicine is on disease, routine incorporation into primary medical care also calls for its inclusion in assessment and promotion of wellness and health. Currently, health promotion programs utilize primary preventative measures such as exercise, diet, weight loss, and stress management [20]. Additionally, clinical indicators and risk factors such as blood pressure, glucose, and lipids are being incorporated into screening, and the potential value of combining these with large-scale genomic and molecular measurements have been discussed [21] but not yet assessed in the context of health promotion. Further, use of these measures is on the 10-year agenda for the United States Department of Health and Human Services *Healthy People 2020 *health promotion program [22].

The Center for Health Discovery and Well Being (CHDWB) is a joint initiative of Emory University and the Georgia Institute of Technology, which has the objective of assessing whether comprehensive annual health evaluation combined with regular discussions with a 'health partner' (an individual trained to interpret clinical profiles and coach on health-related behavior) can help people make more informed and better personal health decisions that maintain wellness, and potentially reduce morbidity and medical treatment for chronic disease [23,24]. In this program, we obtaincomprehensive clinical data pertaining to metabolic, cardiovascular, skeletal, and mental health, we carry out a survey assessment of nutrition, behavior, and family history of disease every 6 to 12 months, and we are performing WGS and other deep genomic profiling for a subset of participants.

The objective of this report is to show how these two types of analysis, namely clinical and genomic, can be considered as complementary views of participant health. Clear instances of agreement and of discordance are described, and strategies for conditioning genomic risk assessment on clinical data are considered. We conclude with a discussion of how the complex and voluminous quantity of data might in the near future be distilled to support actionable medical inference and personal lifestyle choices.

## Methods

### Subjects

Eight Caucasian individuals (four men and four women) were selected from a longitudinal cohort of healthy adult volunteers at the CHDWB at Emory University Midtown Hospital (Atlanta). The CHDWB participants were broadly representative of Emory employees and were free of any known acute illness at the time of recruitment. The eight selected individuals are drawn from a panel of 500 CHDWB participants who had completed at least three visits during the first 2 years of the Center's existence, and were chosen pseudo-randomly to represent a range of diversity for metabolic and cardiovascular phenotypes (for summary, see Additional file 1). The eight individuals were a non-random sample in the sense that they were selected from the upper or lower deciles for body mass index, percentage body fat, high-density lipoprotein cholesterol (HDL-C), and triglyceride levels, and the Beck Depression Index and Augmentation Index values were used to capture slightly different clinical profiles. They thus represent classically 'fit' and 'unfit' phenotypes, whichwere nevertheless different with respect to blood fat and sugar. The individuals were a random sample in the sense that another 30 individuals with similar profiles could easily have been chosen. Their Framingham Risk Scores (FRS) for diabetes and cardiovascular risk were distributed across the observed range in the entire cohort, as are their genotypic risks for both diseases (see Additional file 2).

### Ethics approval

The study was performed in accordance with the Declaration of Helsinki. It was approved by the institutional review boards (IRBs) of Emory University (IRB00007243) and the Georgia Institute of Technology (H09364) for collection of clinical and genomic data following written consent, and although we discussed openly with participants regarding their clinical data, approval for provision of genetic results to individual participants has not yet been sought or provided. Because of this, in the interests of participant privacy, data in some figures and tables are presented as z-scores so as to preclude personal identification. We carried out analyses (see Additional file 3) to confirm that participants would not be able to identify themselves unambiguously from the data presented here, as most have clinical profiles that are very similar to those of other individuals in the study. In addition, rare variants were for the most part excluded from discussion, as the IRB considers these to be more potentially disturbing in the absence of professional consultation were an individual to suspect that they are represented.

### Clinical assessments

Details about the recruitment of participants and collection of biomedical and health status data at CHDWB have been described previously [24]. Participants underwent extensive clinical measurements to assess their health status at study initiation, and 6 and 12 months later, and most are continuing to participate with annual evaluations. Data gathered include anthropomorphic measurements, laboratory tests including complete blood counts, metabolic and lipid profiles, urinary and serum biomarkers for oxidative stress, inflammation, and immune function, pulse wave velocity assessment of cardiovascular function (SphygmoCor; AtCor Medical, Sydney, Australia), whole body densitometry, and assessment of mental and behavioral health (NexAde; NexSig Neurological Examination Technologies Ltd, Herzliya, Israel) as described previously [24]. Self-reported family and personal medical histories were also recorded, along with extensive online surveys that were filled in at the participants' convenience at or around the time of each visit. Blood samples were collected at each visit for all the participants, and DNA was extracted from buffy coats isolated at first visit.

Risk predictions for 8 year risk of diabetes and 10 year risk of cardiovascular diseases were calculated using the equations provided by the Framingham Heart Study [25] online [26]. We derived z-scores for continuous clinical variables using the entire CHDWB dataset of over 500 individuals by subtracting the mean and dividing by the standard deviation. The mean of the first three visits was considered in all assessments reported here.

### Whole genome sequencing

WGS was performed by the Illumina Genome Sequencing Network at the University of Washington on HiSeq2000 (Illumina Inc., San Diego, CA, USA) automated sequencers. Briefly, 100 μl of genomic DNA (> 60 ng/μl concentration) was sheared to give a mean fragment size of 500 bp, and sequencing libraries were generated. Imaging and analysis of 100 bp paired-end read data was performed using standard Illumina software. Approximately 125 billion bases that passed the Illumina analysis filter were obtained for each genome. Mean non-N reference (that is, after excluding gaps) coverage was approximately 36X, with 95.5% (mean) of the positions having coverage of at least 10X. The genome sequences were aligned against the Human Reference Genome assembly (hg19 sequence) using CASAVA (Consensus Assessment of Sequence And Variation) software (Illumina). On average, 87% of each individual's quality filtered reads were aligned. High-confidence variants with a quality score above 20 were retained. The accuracy of the generated genome sequences was confirmed by comparison with previously determined genotypes from Illumina OmniQuad arrays, which showed over 99% concordance for all individuals.

### Genetic risk assessment based on common variants

Genetic risk predictions for various diseases were generated using our VARIMED (Variants Informing Medicine) database of complex disease associations [15], and our previously reported pipeline for combining odds ratios (ORs) of robustly associated single-nucleotide polymorphisms (SNPs) with diseases and traits [27]. An individual's genetic risk for a disease was calculated as their 'post-test probability'. We first computed likelihood ratios (LRs) for each SNP as the ratio of the probability of the genotype in an affected person to that of an unaffected person. LRs for each locus were computed from each case-control study be dividing the genotype frequency in cases by the frequency in controls, weighted by the sample size of the study. Thus, for an individual with genotype *g *in SNP *x *found in *i *= 2 ...s studies, each of size *S*(*i*), the LR is computed as:

$$\text{log}\;LR\left(x\right)=\frac{\sum_{i=1}^{s}\text{log}\frac{F\left(g\;in\;cases\right)}{F\left(g\;in\;controls\right)}\times \sqrt{S\left(i\right)}}{\sum_{i=1}^{S}\sqrt{S\left(i\right)}}$$

Only loci that have been found in GWAS in individuals of European ancestry in at least one study with *P *< 1 × 10^-6 ^were used in estimating LR, using only the most significant site in a haplotype block (*r*^2 ^≥ 0.8). Next, all LRs were combined with pre-test probabilities, namely the baseline lifetime risk for disease, to estimate the post-test probability [27]. Sex-appropriate pre-test probabilities estimated from published reports were used to estimate the post-test probabilities by converting them to pre-test odds using the formula pre-test probability/1-pre-test probability, multiplying by the genotypic LR to give the post-test odds, and then converting these to post-test probability by dividing the post-test odds by 1 + post-test odds.

For example, for *n *number of SNPs, the post-test odds are given by:

$$\text{Post - test}\;\text{Odds}\;\text{ = }\;\text{Pretest}\;\text{Odds}\;\text{*}\;\overset{n}{\underset{i=1}{\prod }}LR\left(i\right)$$

### Clinical risk assessment

**Figure 1:**
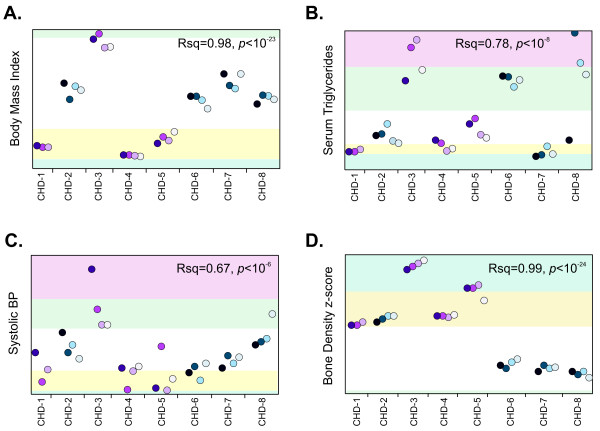
**Temporal change in clinical measures in the eight Center for Health Discovery and Well Being (CHDWB) participants: (A) body mass index (BMI), (b) serum triglycerides, (c) systolic blood pressure (SBP), and (d) bone mineral density (BMD)**. Each plot shows the temporal sequence from first to fourth visit over 3 years (left to right, dark to pale shading) for the four men (blue) and four women (purple) in the study. Data were only available for the first three visits for CHD-1. Adjusted R^2 ^and significance measures refer to ANOVA of the individual differences, providing an estimate of the proportion of variance between individuals for each measure. Background color coding shows very high (pink), high (green), intermediate (white), low (yellow), and very low (blue) risk score categories defined by 1 or 2 standard deviation units from the CHDWB mean. These categories are also used in Figure 3 (see text for specific results). In order to protect participant privacy, actual trait values are not shown, but ranges in the full cohort are 16 to 61 for BMI, 30 to 708 mg/dL for serum triglycerides, 78 to 187 for SBP, and -3 to +3 for BMD z-scores.

Each person was classified according to five levels of risk (very low, low, intermediate, high, or very high) for a disease/trait according to whether they were in the upper or lower one or two standard deviation unit bins of measured clinical markers for that disease/trait in the entire CHDWB cohort (Figure 1). Clinical risk was assessed in eight major disease categories: immunological, metabolic, cardiovascular, musculoskeletal, respiratory, cognitive, psychiatric and oncological. A list of diseases and the measured clinical attributes considered in each category is provided (see Additional file 4), but note that this list will vary depending on the range of clinical attributes that are available in any study or clinic. The strategy is simply to average multiple clinical measures so as to place individuals in five bins for each category. We are currently evaluating computational strategies for combining the scores in a weighted manner that also accounts for co-variance, but in this study we used the simpler strategy of simply averaging each of the contributing z-scores. Additional categories might include organ failure and reproductive health, but we did not have data pertaining to these at this time.

### Integration of genetic and clinical data

For joint clinical and genetic risk assessment, we describe two exploratory approaches.

**Figure 2:**
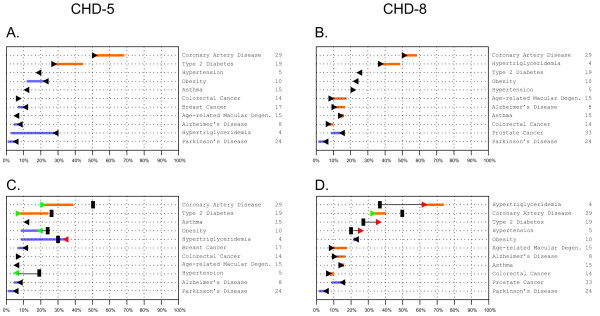
**(A,C) Risk-o-gram plots for common diseases in standard format for two individuals, (A) CHD-5 and (B) CHD-8**. (C,D) Risk-o-grams adjusted according to observed clinical risk for (C) CHD-5 and (D) CHD-8. Gender and age-specific disease prevalence for Caucasians are indicated by the black triangles (or hash marks in lower panels). Genotypic effects are predicted to increase (right point) or decrease (left point) overall risk by the indicated magnitude (orange or purple, respectively), resulting in the indicated rank-ordered overall risk. The number of single-nucleotide polymorphism (SNPs) used in the computation for each disease is indicated to the right. (C,D) The adjusted risk-o-grams according to observed clinical risk either increase (red triangle) or decrease (green triangle) the baseline without affecting the genotypic component, but result in adjustment of overall risk and rank order. Note that CHD-5 is a woman, and CHD-8 a man, so breast and prostate cancer are indicated for each as appropriate.

The first approach directly matches GWAS results with individual diseases. Two limitations to this approach are either that there are no appropriate clinical biomarkers for some diseases in our cohort (such as for asthma and cancer), or that some biomarkers are precisely the endophenotype of the disease/traits investigated in GWAS (for example, triglycerides for hypertriglyceridemia or body mass index (BMI) for obesity), so in a sense the two are redundant. Nevertheless, we proceeded to use the strategy shown in Figure 2 for five inter-related and prevalent conditions: CAD T2D, hypertension, obesity, and hypertriglyceridemia. There are two analytical issues, namely assessing each person's relative risk, and adjusting the post-test probability based on that risk. For CAD, T2D, and hypertension we computed FRS for each person at each visit across the entire CHDWB database. These scores were averaged over the first three visits to generate an individual's average FRS, which was divided by the sample mean to generate the LR. We used the relationship that post-test probability = pre-test probability × LR/(1 + (pre-test probability × (LR-1))) to generate an adjusted baseline, which can then be modified by genotypic risks. For obesity and hypertriglyceridemia, the intention is to show an individual whether their risk is due to a combination of both clinical and genetic factors. For instance, individuals with incident obesity or hypertriglyceridemia have the disease, but it is nevertheless possible to report risk factors of less than 100%. We proceeded by noting that the relative environmental and genetic contributions are reflected in the heritability, which can thus be used to scale the clinical contribution as a proxy for the environment. Estimates vary in the literature, but here we assumed heritability of 50% for obesity and 30% for hypertriglyceridemia. Each individual's z-score was computed and the LR for individuals with the same clinical z-score was identified. The pre-test probability was multiplied by 2 × *h^2 ^*× *LR *(namely the LR for obesity or 60% of the LR for hypertriglyceridemia), providing a newly scaled pre-test probability that then seeded the genotypic adjustment.

**Figure 3:**
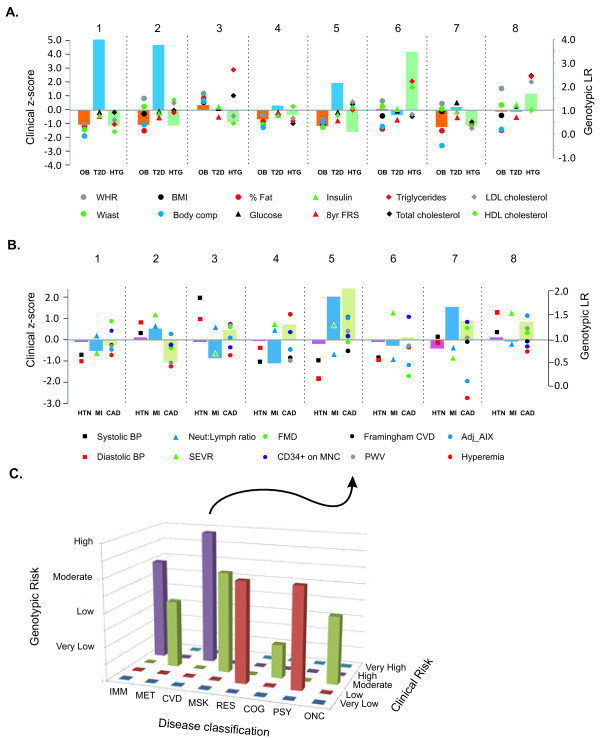
**Joint representation of clinical and genetic risk assessment**. (A) Metabolic risk showing joint genotypic likelihood ratios for obesity (OB), Type 2 diabetes (T2D), and hypertriglyceridemia (HTG) as bars, and related clinical measures as the indicated points, for each of the 8 individuals in the study. (B) Similar cardiovascular risk assessments for hypertension (HTN), myocardial infarction (MI), and coronary artery disease (CAD) as bars, along with related clinical measures and/or Framingham Risk Score. All measures were averaged over the first three visits to the Center for Health Discovery and Well Being (CHDWB). (C) Proposal for 'gridiron plot' representation of clinical risk (y-axis) against genotypic risk (z-axis) in eight disease domains described in the text, for individual CHD-5. The plot gives a glimpse of where the two types of assessment are concordant (for example, cardiovascular disease (CVD), cardiovascular)or discordant (that is, immunological (IMM)), and more refined analyses such as in (A) and (B) provide further clues as to the genetic basis of overall risk.

The second approach combined multiple clinical and genetic measures in order to generate an overall portrait of risk in the eight major disease categories mentioned above (Figure 3). The z-scores for clinical parameters were adjusted with respect to risk predisposition: for traits that are known to confer risk at a lower level (such as HDL-C, hyperemia) we plotted the additive inverse. Similarly, each participant's genetic risk score was ranked according to percentiles into five categories. The 'gridiron plot' (Figure 3C) then showed the relationship between estimates, and allowed an individual to immediately see for which classes of disease they have an increased, reduced, or discordant risk. They could then consult individual clinical and genetic measures (Figure 3A, B) to discover exactly which attributes they may consider in developing a health action plan.

## Results

### Clinical and genomic measures of eight participants in the CHDWB

In general, clinical parameters show much higher inter-individual than intra-individual variance, shown in Figure 1 by the progression of values over time (left to right) for four traits in each of the eight individuals in the study. Trends were in a hopeful direction for several of the participants [23]. For example, BMI dropped consistently for the most overweight individual (CHD-3, Figure 1A). Occasionally, one or more individuals showed hypervariable measurements over time (triglyceride levels for CHD-8; Figure 1B), but for the purposes of clinical risk evaluation, average values reflect the rank order of individuals, and are sufficient to place individuals in broad categories, from very low (light blue shading) to very high (pink).

SNP, indel and copy number variant (CNV) or structural polymorphism called by the Illumina CASAVA algorithm, as well as the number of homozygous coding variants and predicted splice site variants. are summarized in Table 1. Each individual had an average of almost 3.7 million SNPs, 623,000 indels, and 4,100 CNVs, consistent with published estimates of polymorphism from WGS [28,29] also summarized in the Table.

**Table 1:** Summary of variations in genome sequences of eight Caucasian subjects, with data from two previously reported studies[27,40].

Sample ID	Total number of variants (>q20)			Coding variants^a^							
				
	SNP	Indel	SV	Number of SNPs				Indel			SV
				
				Synonymous (rare homoz)	Missense (rare homoz)	Nonsense	Splice overlap	FS	NFS	Overlap^b^	
CHD-1	3,722.234	641,792	4197	11,887 (18)	11,434 (29)	64	76	303	299	125	41
CHD-2	3,701.558	639,005	4739	11,842 (11)	11,708 (33)	60	81	334	290	118	37
CHD-3	3,691.270	632,544	4033	11,912 (9)	11,488 (31)	65	71	279	304	116	37
CHD-4	3,691.337	633,475	4114	11.757 (9)	11,457 (25)	56	90	317	280	106	49
CHD-5	3,734.820	645,032	3977	11.929 (9)	11,745 (35)	62	90	343	307	123	43
CHD-6	3,650.690	602,744	3916	11.560 (12)	11,285 (37)	60	80	342	280	112	32
CHD-7	3,643.046	597,363	4011	11.814 (17)	11,480 (41)	61	85	289	287	109	31
CHD-8	3,647.944	590,064	3828	11.619 (9)	11,255 (18)	54	76	311	281	95	38
Pelak *et al. *[28]	3,473.639	609,795	805 (CNVs)	-	11069	117	99	479	898	-	-
Shen *et al. *[40]	3,307,678	421,088	-	9,612	9,082	87	-	217	164	-	-

Abbreviations: CNV: copy number variant; FS: frameshift; NFS: non-frameshift; SV: structural variant.

^a^The coding variants were classified based on Gencode version 7.

^b^Overlap: located within 2 nucleotides of the exon-intron boundary.

### Genotypic risk prediction

Genotypic risk assessments were generated for each of the participants, and are presented as 'risk-o-gram' plots (see Additional file 5). Baseline pre-test odds were simply taken from published epidemiological data on gender, age, and ethnicity-adjusted disease prevalence [15]. The number of SNPs per disease or trait ranged from 1 to 66 (for Crohn's disease), with an average of 9 and median of 4, where 25 conditions were evaluated from at least 10 SNPs. Representative short risk-o-grams are shown for only the common diseases for two individuals on the linear scale (Figure 2).

Because not all the individuals within a population of the same sex, age, and with the same environment have the same overall risk, we propose that pre-test odds can be conditioned on an individual's clinical profiles. These partially capture the lifetime of individual-specific genetic and environmental factors that have shaped that individual's health status. An intuitive way to perform this conditioning is to modify the baseline risk using a multiplier that is a function of the heritability, along with each individual's z-scores for relevant clinical parameters that are related to the specific disease.

A simple implementation of the approach resulted in the further modified risk-o-grams (Figure 2C,D). In this case, there was no change in relative genotypic risk for a given disease, but the absolute risk predictionswere in some cases modified substantially, and as a result of this, the rank order of diseases changed. For example, the hypertension and hypertriglyceridemia profiles were reversed for individual CHD-5 on the left, because the clinical data implies low blood pressure but high serum triglycerides, despite the currently understood genetic risk. Triglyceride levels were even higher than expected for CHD-8 on the right, which contributed to a higher T2D FRS, but other factors have moderated this individual's CAD risk. Our representations (Figure 2) are provided as illustrations of the principle of how genotypic risks can be adjusted according to clinical status, and should be interpreted in light of the numerous methodological challenges that need to be addressed. Further research is required to refine the implementation, and establish whether or not this will or should affect an individual's health behavior choices and/or future clinical outcomes.

### Comparison of clinical and genotypic risk assessments

An alternative to computing overall disease probabilities by combining both genotypic and clinical LRs is to report both evaluations in a simple graphical manner (Figure 3). Commercial providers of personal genome services currently present results in browsable disease-by-disease or gene-by-gene formats, which do not lead themselves to data integration, and arguably either overwhelm individuals or focus attention on a few key results. By contrast, our proposal is to present participants with summaries of their risk factors and biological indicators across different aspects of wellness. We developed this notion in the form of a gridiron plot of risk (Figure 3C) assessed in the aforementioned eight domains of health along the x-axis, from both clinical (y-axis) and genotypic (z-axis) data. These eight proposed domains reflect concerns that most relatively healthy middle-aged people have about joint and back pain, body weight, infections, and irritability or sleep deficits, few of which are directly measured by GWAS studies, yet can conceivably be related to GWAS disease results. These domains correspond to established categories used to classify disease, such as those in the International Classification of Diseases (ICD), but a more refined classification might be based on the human disease network built around shared genetic etiology [30]. Additional domains can be considered, such as reproductive health, eyesight and hearing, and renal health. These domains are most likely to be of concern for subscribers to wellness and/or preventative care programs, such as the CHDWB, in which health maintenance and disease prevention take medical priority.

One individual, CHD-5, (Figure 3C) is a very fit Caucasian woman in her 50s. There was no indication of great concern for the musculoskeletal, respiratory, cognitive, psychiatric and oncological domains, but interesting findings were suggested by the first three domains to the left of the gridiron. Her metabolic profiles showed general concordance, as her low BMI and low percentage of body fat, were matched by her genotypic risk scores for metabolic disease and T2D in the low to moderate range. There was also clear concordance between her clinical and genotypic profiles in the cardiovascular disease (CVD) domain, as she was shown to have moderately high risks of CAD, myocardial infarction, and stroke (each approximately two-fold ORs combined from a total of 80 SNPs), and her standard measures of cardiovascular health (augmentation index of arterial stiffness, hyperemia, measure of peripheral oxygenation) both placed her in the high-risk category, contrary to the Framingham risk assessmentthat generated her revised estimate in Figure 2 (black dot). In the immunological domain, she had few indicators of immune impairment other than self-reported intestinal complaint, so it is interesting to note her genotypic risk for ulcerative colitis was relatively high, whereas that for Crohn's disease was low. The utility of the gridiron plot is that it is designed to help the individual pay attention to specific aspects of their health. A drawback of the necessarily superficial summarization is that conflicting genotypic (or clinical) assessments within a domain can cancel each other out. More detailed representation of how the data types can be combined and presented in each domain, across multiple individuals, is shown (Figure 3A, B).

Perusal of Table 2 indicates many examples where common variant risk evaluation was concordant with clinical data (that is, the two types of risk are in the same direction), with an approximate 2:1 ratio of concordance to discordance. For example, for the four individuals with consistently low weight and BMI measures (CHD-1, CHD-4, CHD-5, and CHD-7) were all found to have a genotypic risk for obesity that was below the population average (see also Figure 3A), although the overweight individual CHD-3 had a slightly increased genetic risk for obesity. For triglycerides, the evaluation was split, with two of the three individuals with very high triglyceride levels (CHD-6 and CHD-8) also showing a greatly increased genetic risk, whereas CHD-3 showed a reduced genetic risk. There were no diabetic subjects in the sample, but the two individuals with very high T2D risk had normal fasting glucose and insulin levels at the time of assessment. For CAD (Figure 3B), the three individuals with the highest genotypic risk had variable clinical profiles: their FRS for CAD were not particularly high, and two of them were at the opposite ends of the augmentation index and hyperemia score ranges (CHD-5 being at high risk, and CHD-7 at low risk, by both criteria), while the third had intermediate clinical CAD-related scores. The results for hypertension were less concordant, possibly owing to the small number of variants considered, but it is noteworthy that the individual with very high blood pressure (CHD-3) had no obvious genetic risk. This may be a case where this individual's lifestyle is a major component of her risk, and notably her systolic blood pressure has dropped consistently over the first 2 years that she has been in the CHDWB program (Figure 1C). Note that these analyses only include highly significant genotypes from GWAS that have been independently replicated, thus they capture a minor proportion of the suspected genetic variance, and consequently there is not a strong expectation at this stage of genomic medicine that the relatively small genotypic samples should be predictive [31,32].

**Table 2:** Genetic predictions and clinical phenotypes related to metabolic and cardiovascular disorders

Sample ID	Common variant prediction	Clinical phenotype	Clinical risk factors
CHD-1	T2D (4.5)	-	FMD, CD34+ cells
CHD-2	T2D (3.8); MI (1.24)^a^	-	BP, N:L ratio, SEVR, HDL-C
CHD-3	Stroke (2.18); CAD (1.21) ^a^; obesity (1.23)^b^	Obese	Total cholesterol, TG, BP, N:L ratio, FMD, PWV, FRS (CVD)
CHD-4	T2D (1.20); CAD (1.32) ^a^	-	SEVR, N:L ratio, hyperemia, CD34+ cells
CHD-5	CAD (2.09)^a^; T2D (2.24); stroke (2.18); MI (1.91)^a^	-	Total cholesterol, LDL-C, SEVR, hyperemia, AIX, PWV
CHD-6	Hypertriglyceridemia (3.48)^b^; atrial fibrillation (1.35)	Hypertriglyceridemia	HDL-C, SEVR, CD34+ cells
CHD-7	Stroke (2.18)^b^; MI (1.70); atrial fibrillation (1.35); CAD (1.40)	Stroke	FMD, CD34+ cells
CHD-8	Hypertriglyceridemia (1.72)^b^; CAD (1.38)^a^	Hypertriglyceridemia	WHR, glucose, FRS (T2D), total cholesterol, LDL-C, BP, AIX, PWV, FMD

Abbreviations: AIX: Augmentation Index; BP: blood pressure; CAD: coronary artery disease; CVD: cardiovascular disease; FMD: flow-mediated dilation; FRS: Framingham Risk Scores; HDL: high-density lipoprotein cholesterol; LDL-C: low-density lipoprotein cholesterol; MI: myocardial infarction; N:L: neutrophil:lympocyte; SEVR: sub-endocardial viability ratio; T2D: Type 2 diabetes; TG: triglycerides; PWV: pulse wave velocity;. Numbers in brackets indicate LR due to genetic variants. ^a^concordance with clinical risk factors, ^b^concordance with clinical phenotype.

### Individualized evaluations

Individual CHD-1 is a woman in her 50s who might be described as 'super-fit' with a body fat percentage under 20% and one of the highest maximal oxygen consumption (VO_2max_) estimates in the entire cohort. Her only current health concern at the time of assessment was recurrent bladder or kidney infections for which she takes medications, but nothing in her genetic profile pointed to this condition. She had increased common variant predictionsfor T2D and age-related macular degeneration, and a slight increase in CAD-related traits. She is homozygous for the *ApoE2 *genotype (present in <1% of Caucasians), which is protective against late-onset Alzheimer's disease, but leads to type III hyperlipoproteinemia in 2% of cases, thereby increasing risk of atherosclerosis [33].

Individual CHD-2 is a man in his mid-40s, also with a very high fitness level, although his low-density lipoprotein cholesterol (LDL-C) levels are toward the upper end of the range typically observed in healthy people. He had some digestive concerns, and an increased T2D prediction. While depression was suggested by his genotype (although very little of the variance in the population for depression is explained by common variants at this time) and his siblingsare affected by depression, his mental health score shows no sign of depression or anxiety.

Individual CHD-3 is a woman in her 60s who has high blood pressure and cholesterol, is classified as obese, and has had cancer. Her risk-o-gram was concordant with obesity, CAD, and stroke, and she is also apparently at increased risk for asthma, Parkinson's disease, Alzheimer's disease, and Paget's bone disease, among others. She appears to be someone for whom genomic medicine alongside longitudinal clinical profiling could have important implications for health maintenance.

Individual CHD-4 is another very fit woman in her 40s, whose primary health concern is allergies, which run in three generations of her family. Like many in the study, she takes supplements for heart and bone health, which may offer some protection against her increased risk of CAD and Paget's disease from common variants. She has a family history of cancer, and increased breast cancer risk was indicated genetically, so careful surveillance may be advisable.

Individual CHD-5 is a woman in her late 50s, whose profiles are highlighted in Figure 5. We observed strong concordance of cardiovascular genetic and clinical risk, as well as a history of intestinal issues that would be consistent with a genotypic liability to ulcerative colitis. However, her genetically increased T2D risk not indicated by her excellent fitness, low BMI, and normal clinical indicators of diabetes. Two rare variants (not shown) suggest visual impairment and color vision deficiency, but there is no indication that these are issues for this woman.

Individual CHD-6 is a relatively younger man in excellent health except for triglyceride levels at the high extreme for the entire cohort, which is consistent with the very strong genetic prediction of hypertriglyceridemia from his common variants.

Individual CHD-7 is a man in his early 60s whose most distinguishing clinical feature was that he had the lowest triglyceride levels in the cohort, and he also had remarkably low signs of inflammation, in that his interleukin (IL)-6, IL-8, and tumor necrosis factor-α levels were all in the bottom few percent of the CHDWB sample, and his neutrophil-to-lymphocyte ratio was also low. Deep analysis of his genome may be revealing with respect to the mechanisms responsible for his low level of inflammation.

Individual CHD-8 is a man in his 40s who was discordant for multiple indicators of heart disease, including high diastolic blood pressure, arterial stiffness, and serum lipids, combined with a high FRS for CAD. These were only mildly indicated by common variant evaluation, but rare homozygous variants have been linked to cardiomyopathy and to carotid stenosis or thromboembolism. Alcoholism was reported in his family, so this person certainly is a good candidate for careful clinical and possibly genetic consideration in development of his health behaviors.

## Discussion

It is inevitable that genome sequence information will be incorporated into individualized medical care over the next couple of decades, but just how it will be utilized remains to be seen. A spectrum of applications ranging from explanation of rare conditions at or before birth to enhancement of medical interventions is likely, and to some extent, genome-wide data may be used to predict and potentially help prevent early onset of chronic disease. Clinicians already utilize family history and clinical information for disease prognosis and diagnosis in a similar manner, while recognizing that these are also not individually definitive indicators of the likelihood of disease progression. Family history and polygenic genotype scores from SNPs identified by GWAS have similar predictive ability for common diseases, but genotypes already outperform family history for many rare conditions [34].

The holistic 'total evidence' approach to integration of clinical and genetic factors in medical evaluation will surely see dramatic improvements in the near future, and will be advanced by developments in several aspects of genomic risk assessment. First, there is ample room for improvement of baseline risk assessment. In Figure 2, we proposed that adjustment of the population prevalence by clinical status has the potential to directly integrate genetic and clinical risk prediction. We emphasize that more research needs to be carried out before this strategy can be considered to be robust, and that medical utility remains to be demonstrated. Note that the OR approach to computation of genetic risk is just one of several methods that could be used. It improves on simple allelic sum measures through the incorporation of allele frequency and effect sizes in the computation, but does not account for epistatic or genotype × environment interactions, and it is not yet clear how well it captures the actual distribution of genetic liability for common disease. In addition, there are important theoretical issues surrounding the computation of genetic risk [35,36], particularly in populations of mixed ancestry. Most importantly, GWAS have as yet discovered only a minority of the variants that contribute to any given disease, in most cases explaining no more than 15% of the risk. This amount of explained variance does not translate into significant risk prediction, for example by receiver operating characteristicanalysis [37], although there are reasons to believe that it does classify individuals who are toward the tails of the distribution. Even in the presence of complete knowledge of the genetic contributions, risk prediction is limited to the square root of the heritability, but we emphasize that none of the scores available to date approach this limit. As the sample sizes of GWAS continue to increase to hundreds of thousands of cases for more common diseases, expanding discovery from dozens to hundreds of loci, genotypic risk assessment will certainly improve [38].

This study was conceived as a pilot investigation of how WGS may be utilized in the context of health maintenance. Participants in the CHDWB interact regularly with a health partner who helps them to interpret their clinical profiles in the context of their own medical issues, and to develop a health action plan [24]. These typically focus on diet, exercise, and stress reduction, but can include specific attention to issues such as low bone density, high blood pressure, or loneliness, and/or lead to physician referral for indications of previously unrecognized heart or metabolic disease. Across the full cohort of almost 700 participants, there are encouraging trends toward improved wellness [23,24], and this is clear for some of the individuals reported here, in terms of significant reduction in BMI and inflammation. It will, however, take prospective and longitudinal studies to evaluate whether wellness genomic profiling is beneficial either to individuals (in terms of maintained wellness) or as a matter of public policy (reduced healthcare costs, improved employee performance).

We do not currently have IRB approval to share the genomic profiles with the participants, so cannot yet evaluate how self-knowledge of gene sequences might also affect health behavior. Instead, we propose a strategy for presenting the diverse data types in a manner that we suspect will help individuals see connections between their genetics, their clinical profiles, and their own health perception. In the short term, the utility of the approach is more likely to be measurable in terms of modification of health behaviors than in economic or life-long health benefits. We show a modified version of Figure 3 (see Additional file 6) that captures the type of data that may be most influential for a hypothetical individual, where an overall view of the health domains is associated with an individual's genotypic and clinical scores relative to the population, along with a list of rare variants of interest. A recent study [39] suggests that clinical geneticists are reluctant to report incidental findings on genetic mutations to patients unless the mutation is known to be pathogenic. However, because the expectations are different in the context of wellness, where subjects actively seek data, we envision that a physician, genetic counselor, or health partner would discuss the summary and appropriate specific details of the evidence with the individual, who would thus be empowered to consider whether they should act upon the genetic eviden.

Clearly, our ability to integrate genomics into health maintenance will improve with experience and the incorporation of more data, including environmental exposures and behaviors. Family history of disease and presence of rare deleterious variants are two obvious types of information that will be highly relevant: risk assessment relative to other family members who have been genotyped will allow a person to evaluate where they stand, given the known sources of variance in the family [16,35], and *de novo *mutations may sometimes explain specific discontinuity between clinical and common variant assessments. We are also gathering data on the metabolome, transcriptome, and epigenome for these eight individuals, and expect these functional genomic data types to provide complementary information that we will evaluate later.

## Conclusion

This pilot study of eight individuals from the CHDWB proposes two approaches for combining and conditioning clinical and genetic profiles, which could facilitate longitudinal evaluation of wellness-focused medical care based on comprehensive self-knowledge of medical risks. The study shows an excess of concordance between genetic prediction and observed sub-clinical disease. Further, we illustrate how more holistic combination of genetic and clinical data can be achieved by visualizing risk in sub-classes of disease. The visualization of concordance and discordance in the genetic and clinical profiles might help develop personalized health action plans in consultation with a health partner. We acknowledge that the data presented here falls short of the gold standards of evidence of inference that are typically required in genetic analysis of causation, but argue that the objective of 'personalized genomics' is not necessarily to predict disease with any certainty, but rather to provide another line of evidence that physicians and other medical practitioners can consider in their interactions with patients. Ultimately, the utility of the approach described here will require prospective evaluation in a cohort of healthy adults followed longitudinally for decades. As the volume of personalized information increases, the issue of who will be responsible for interpreting and explaining the assessments to individuals becomes more acute, and suggests the need for training of a new class of genomic healthcare professional and development of novel ways to present the information.

## Abbreviations

BMI: Body mass index; CAD: Coronary artery disease; CASAVA: Consensus Assessment of Sequence And Variation; CHDWB: Center for Health Discovery and Well Being; CNV: copy number variant; CVD: Cardiovascular disease; FRS: Framingham Risk Scores; GWAS: Genome-wide association studies; HDL-C: HDL-cholesterol; IL: interleukin; IRB: Institutional review board; LR: Likelihood ratio; SNP: single-nucleotide polymorphism; T1D: Type 1 diabetes; T2D: type 2 diabetes; VARIMED: Variants Informing Medicine; WGS: whole genome sequencing.

## Competing interests

AB is a founder and consultant to Personalis, Inc., a genetic testing company. RC is an employee of, and AM is a consultant to, Personalis, Inc. The remaining authors declare that they have no competing interests. Stanford University holds the intellectual property on genotypic risk assessment technologies described in the manuscript that may be licensed to various companies, but the data visualization strategies presented in Figure 3 and Additional File 6 are not intellectual property of Personalis, Inc. The CHDWB also offers fee-for-service clinical assessment and health partner evaluation for a small number of individuals not included in this study.

## Authors' contributions

CJP, AS, and RT performed all of the genome analysis and risk-assessment computations, and wrote the paper with GG. TP analyzed the potential function of amino acid mutations. JZ provided support for genome sequence feature extraction. DA prepared the DNA samples for sequencing. RC and AM provided software, databases, and advice for risk assessment. GM and KB direct the CHDWB, and performed clinical assessments. AJB, CJP, and GG conceived the study approach and supervised all analyses. All authors read and approved the final manuscript.

## Supplementary Material

Additional file 1 **Clinical attributes of the population**. Table showing the clinical attributes of the subjects included in the study at their first visit. Click here for file

Additional file 2 **Framingham Risk Score (FRS) and genotypic risk score for Type 2 diabetes and cardiovascular disease**. The two plots contrast genotypic risk score and clinical FRS for each of 182 people in the Center for Health Discovery and Well Being (CHDWB) cohort. In both cases, the two scores were positively correlated reflecting contributions of both pre-test and genotypic risks to the correlation with Framingham scores. Black dots show the scores for the participants discussed in this paper. Click here for file

Additional file 3 **Non-identifiability of participants on basis of clinical phenotypes**. Two-way hierarchical clustering of z-scores of 40 traits (columns) for 380 participants (rows) at 3 successive visits shows clustering of participants, 3 of whom (indicated by orange, green or red markers to left) cluster separately in at least one visit. The other five participants have clinical profiles that were always most similar to one another, but in most cases were so similar to other participants also that they do not uniquely define a person, given the data reported in this paper. Click here for file

Additional file 4 **Disease categories**. Table showing the classification of clinical phenotypes into various disease categories for clinical risk assessment Click here for file

Additional file 5 **Risk-o-grams**. Figure showing the risk-o-gram plots depicting the genotypic risk for all eight subjects. Click here for file

Additional file 6 **Summary clinical profile**. This one-page summary of the joint genomic and clinical profile for a hypothetical individual suggests how health professionals might present data to patients. The radar plot at the top summarizes health risks for one or more diseases of interest in each of the health domains shown in Figure 3, with the outer ring representing very high genotypic risk and the inner ring very low risk. The size of each point shows the magnitude of clinical risk in the same domain, with green dots highlighting concordant high risk, red dots discordant low genetic and high clinical risk, and blue dots discordant high genetic but low clinical risk. These are shown in more detail below, where the frequency distribution summarizes the genetic risk estimates across a relevant comparison population, and the box-and-whisker plots show the first two standard deviation intervals either side of the mean for associated clinical parameters. Colored points indicate the position of the individual relative to the comparison population. For example, this individual has relatively high genetic risk of depression, which corresponds to high Beck Depression Index, low mental health summary score, and very low social function (possibly suggesting an area for behavioral modification). In the cardiovascular domain, she has very high blood pressure despite low genetic risk of hypertension, and this contributes to relatively high Framingham Risk Score for cardiovascular disease (CVD risk) despite normal arterial stiffness. In the metabolic domain, the data show that she is currently healthy, but a high genetic risk suggests a need for ongoing surveillance. Finally, the report would mention rare variants of various types, including homozygous deleterious alleles that are known to promote rare conditions, or to be protective, as well as carrier status for rare variants that might be of interest in the context of family planning. In addition to this summary report, we envision that a more detailed description of specific findings, including the strength of evidence for associations and any data on clinical outcomes and interventions, would be provided to the patient, and discussed along with appropriate explanation of the genetics and biology. Click here for file
