# Supplementary material for: Whole genome sequencing in support of wellness and health maintenance
Source: Genome Med. 2013 Jun 27;5(6):58. doi: 10.1186/gm462 (PMC3967117; doi:10.1186/gm462)
Supplement: Additional file 1 — Clinical attributes of the population. Table showing the clinical attributes of the subjects included in the study at their first visit. [file gm462-S1.PDF]

Additional file 1: Baseline clinical characteristics of the participants

	**Range**	**CHD 1**	**CHD2**	**CHD 3**	**CHD 4**	**CHD 5**	**CHD 6**	**CHD 7**	**CHD 8**
Gender		F	M	F	F	F	M	M	M
Age (years)	18 - 84	50s	40s	60s	40s	50s	40s	60s	40s
BMI (kg/m^2)	16.6 - 61.2	-1.17	-0.14	0.60	-1.32	-1.13	-0.35	0.03	-0.48
Body fat (%)	8.0 - 59.9	-1.37	-0.90	0.82	-0.87	-0.79	-1.25	-1.42	-1.47
Waist-hip ratio	0.60 - 1.32	-1.33	0.89	0.89	-0.22	-1.11	0.66	0.44	0.89
Systolic B.P. (mmHg)	78.0 - 187.0	-0.09	0.76	3.44	-0.74	-1.59	-0.93	-0.74	0.24
Diastolic B.P. (mmHg)	40.0 - 117.0	-0.58	1.80	2.28	-0.29	-1.53	-1.53	-0.96	1.32
Fitness level	-	Superior	Good	Superior	Superior	Superior	Excellent	Superior	Fair
GSH (uM)	0.59 - 15.23	2.97	0.46	-0.47	1.33	0.24	1.93	1.34	0.87
Cys (uM)	37.7 - 345.3	-0.65	-0.55	1.74	-1.06	-0.01	-0.40	-0.31	-1.54
IL-6 (pg/mL)	0.01 - 37.05	-	-0.04	1.57	-	-0.10	-0.13	-0.45	-0.19
IL-8 (pg/mL)	0.30 - 89.50	-0.02	-0.19	2.28	-0.76	-0.42	-0.38	-1.40	-0.42
TNF-α (pg/mL)	0.01 - 36.2	-0.89	-0.80	-0.26	-0.68	-0.30	-0.67	-1.63	0.66
IFN-γ (pg/mL)	0.01 - 3.51	0.99	1.77	0.54	1.09	0.12	0.57	0.09	-0.71
25-hydroxyvitamin D (nmol/L)	5.0 - 110.0	-0.34	1.35	-0.09	2.19	-0.17	1.26	0.16	0.16
Creatinine (mg/dL)	0.46 - 2.02	-0.39	1.20	0.94	-1.11	-0.98	1.93	0.81	0.61
Glucose (mg/dL)	54.0 - 612.0	-0.14	-0.19	0.31	0.22	-0.14	-0.14	0.31	0.22
Total Cholesterol (mg/dL)	92.0 - 387.0	0.12	0.95	0.92	-0.83	0.92	-0.44	-0.69	1.95
HDL-C (mg/dL)	17.0 - 128.0	1.87	-0.38	0.86	-0.66	-0.21	-1.67	1.53	0.69
LDL-C (mg/dL)	14.0 - 227.0	-0.55	1.41	-0.20	-0.39	1.10	-0.39	-1.22	2.02
Total chol:HDL-C	1.4 - 8.5	-1.26	0.73	-0.37	-0.07	0.53	2.03	-1.36	0.23
Triglycerides (mg/dL)	30.0 - 708.0	-0.99	-0.29	1.99	-0.49	0.19	2.21	-1.16	-0.51
TSH (uU/mL)	0.01 - 21.94	0.09	0.47	-0.11	0.43	-0.10	0.17	-0.11	-0.44

Data presented are Z-scores of measurements at first visit of subjects, calculated using data from CHDWB cohort. Ranges (minimum – maximum) for the traits in the entire CHDWB cohort are provided in conventional units. BMI: body mass index; GSH: glutathione; Cys: cysteine; IL-6: interleukin-6; IL-8: interleukin-8; TNF-α: tumor necrosis factor alpha; IFN-γ: interferon gamma; HDL-C: high density lipoprotein –cholesterol; LDL-C: low density lipoprotein-cholesterol, TSH: thyroid stimulating hormone.
