# Supplementary figures and images for: Whole genome sequencing in support of wellness and health maintenance
Source: Genome Med. 2013 Jun 27;5(6):58. doi: 10.1186/gm462 (PMC3967117; doi:10.1186/gm462)

**Additional File 2.** Patel, Sivadas, et al. 2013

**A.**

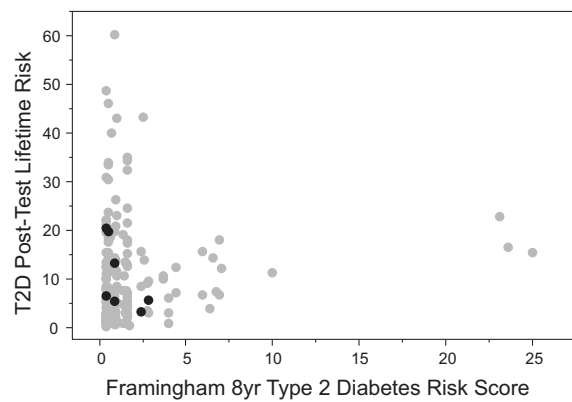

**B.**

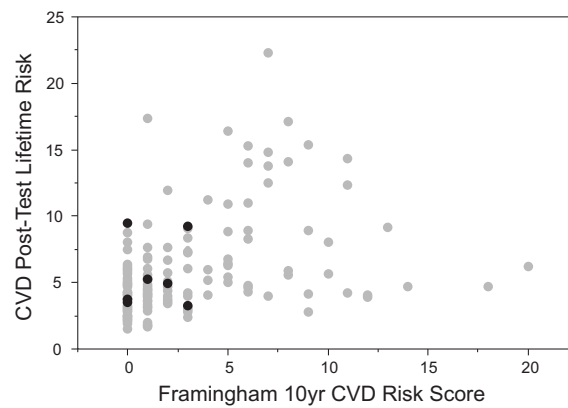

Supplement: Additional file 2 — Framingham Risk Score (FRS) and genotypic risk score for Type 2 diabetes and cardiovascular disease. The two plots contrast genotypic risk score and clinical FRS for each of 182 people in the Center for Health Discovery and Well Being (CHDWB) cohort. In both cases, the two scores were positively correlated reflecting contributions of both pre-test and genotypic risks to the correlation with Framingham scores. Black dots show the scores for the participants discussed in this paper. [file gm462-S2.PDF]

**Additional file 3.** Non-identifiability of participants on basis of clinical phenotypes.

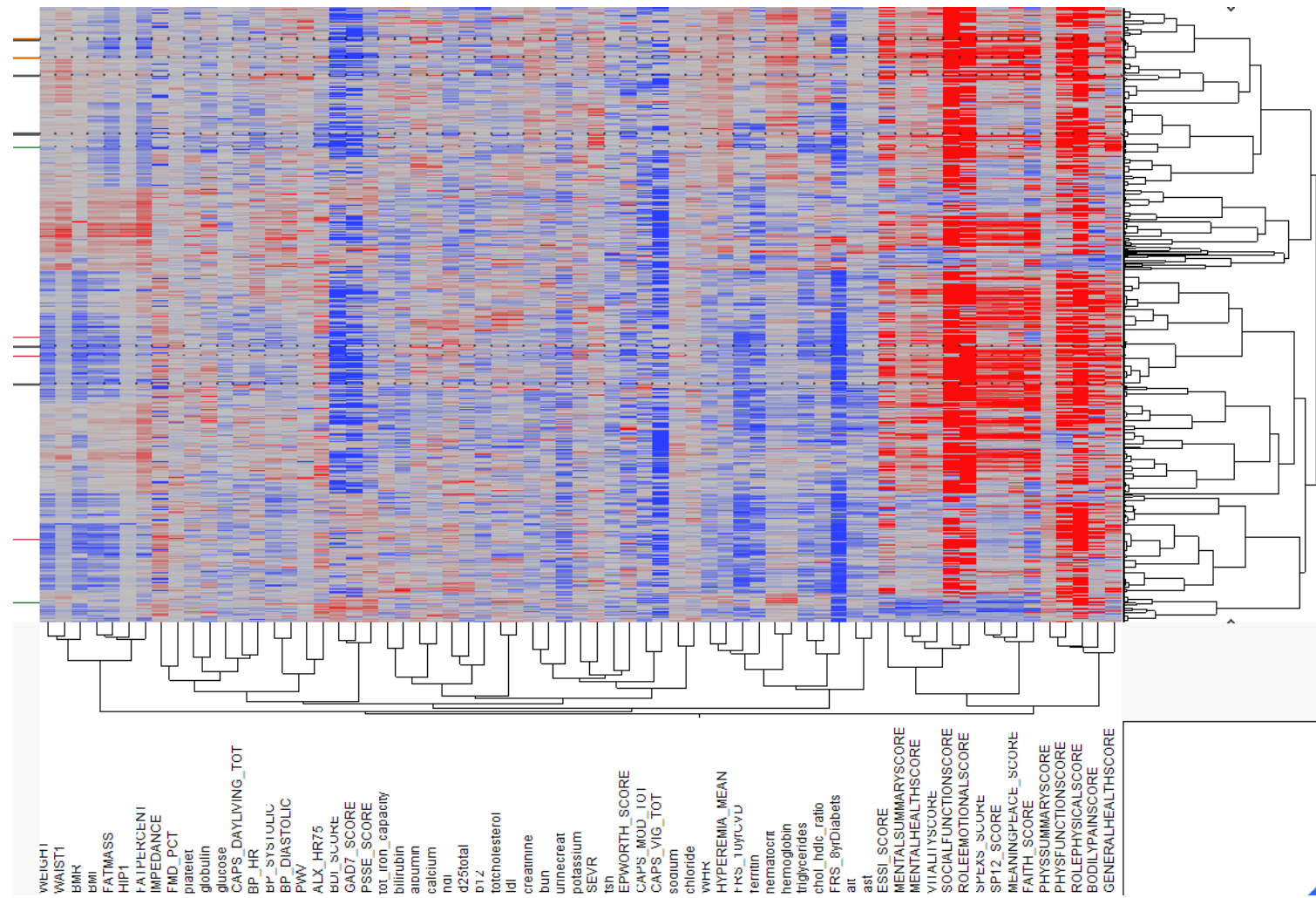

Supplement: Additional file 3 — Non-identifiability of participants on basis of clinical phenotypes. Two-way hierarchical clustering of z-scores of 40 traits (columns) for 380 participants (rows) at 3 successive visits shows clustering of participants, 3 of whom (indicated by orange, green or red markers to left) cluster separately in at least one visit. The other five participants have clinical profiles that were always most similar to one another, but in most cases were so similar to other participants also that they do not uniquely define a person, given the data reported in this paper. [file gm462-S3.PDF]

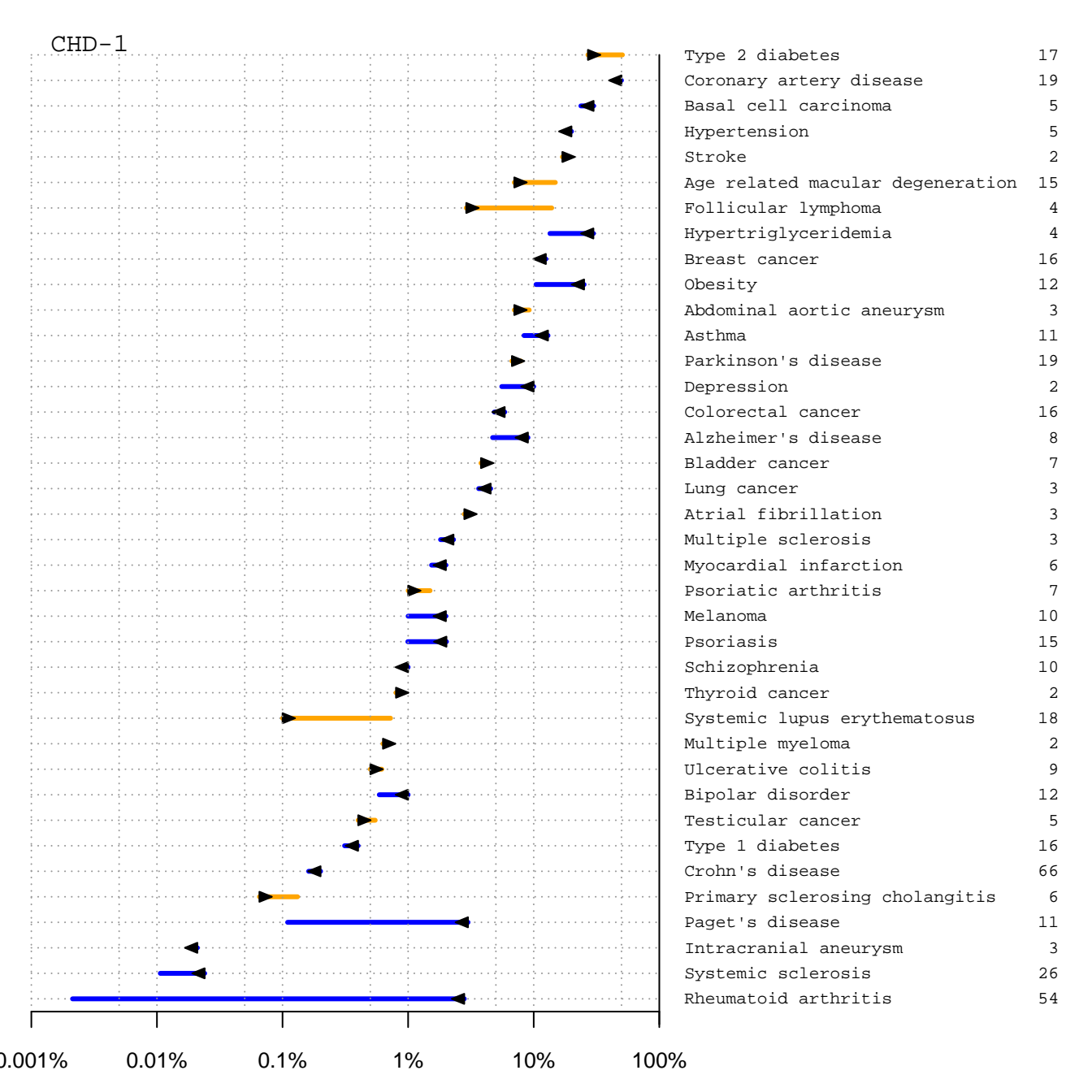

CHD-2

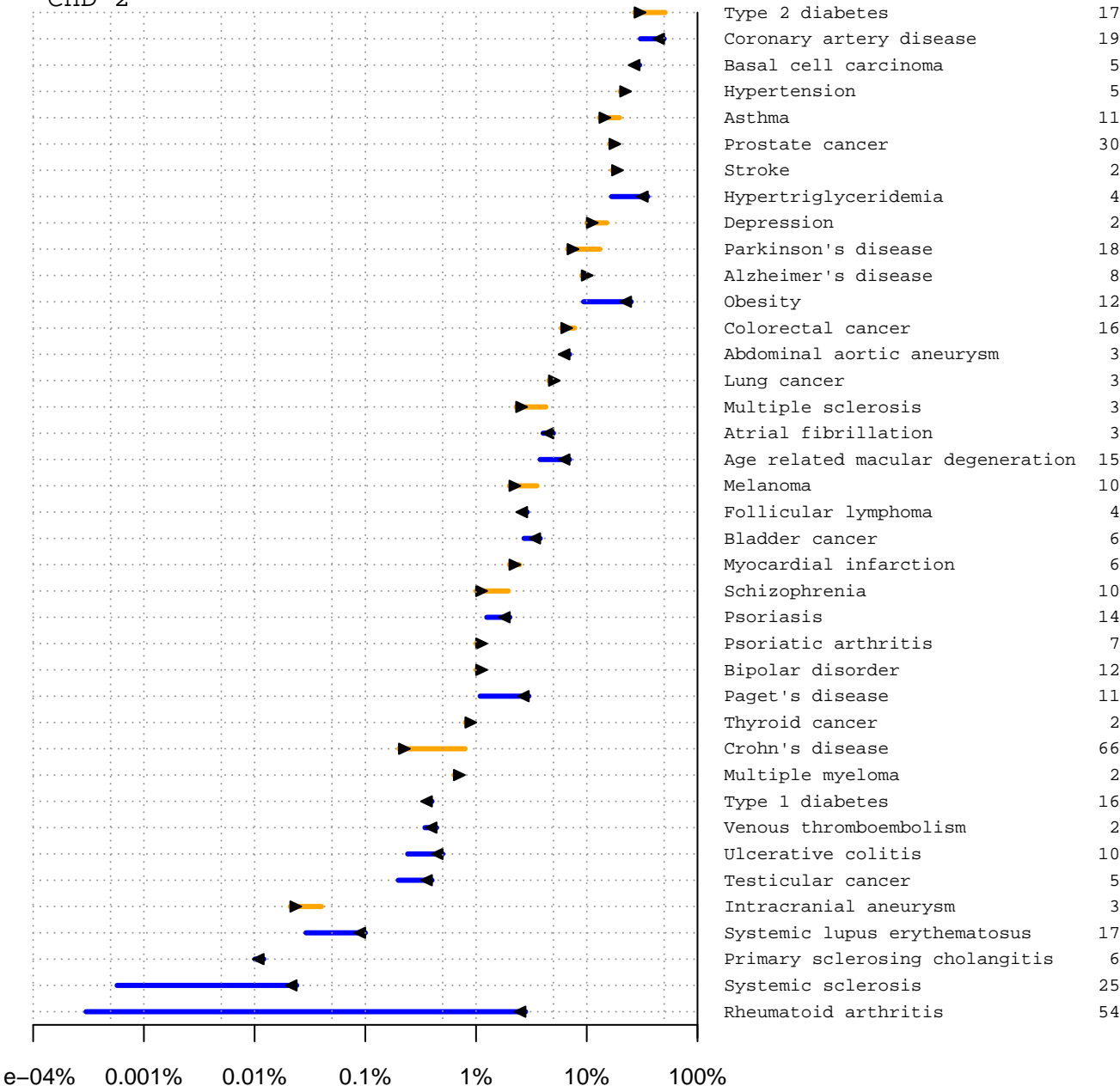

CHD-3

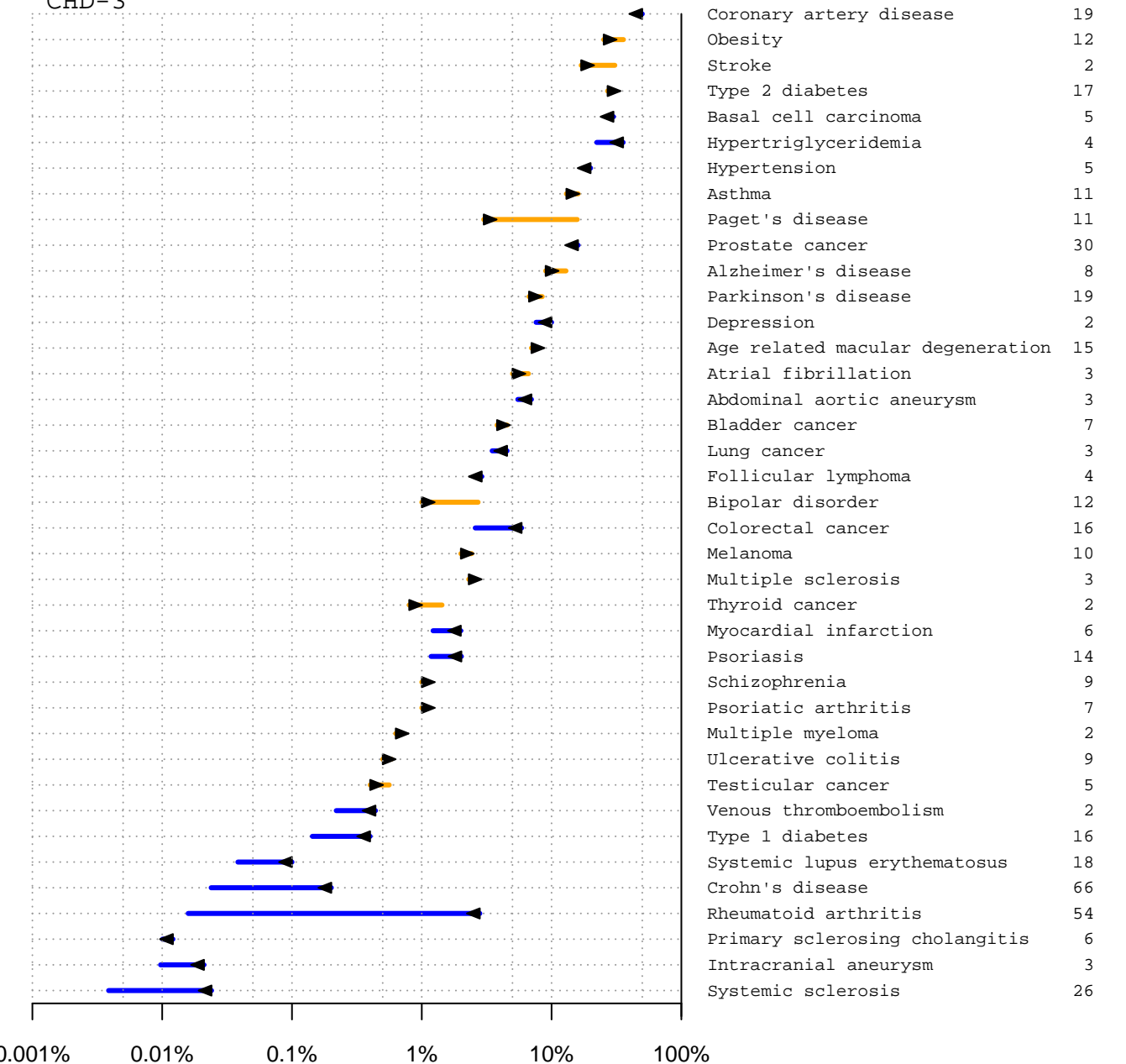

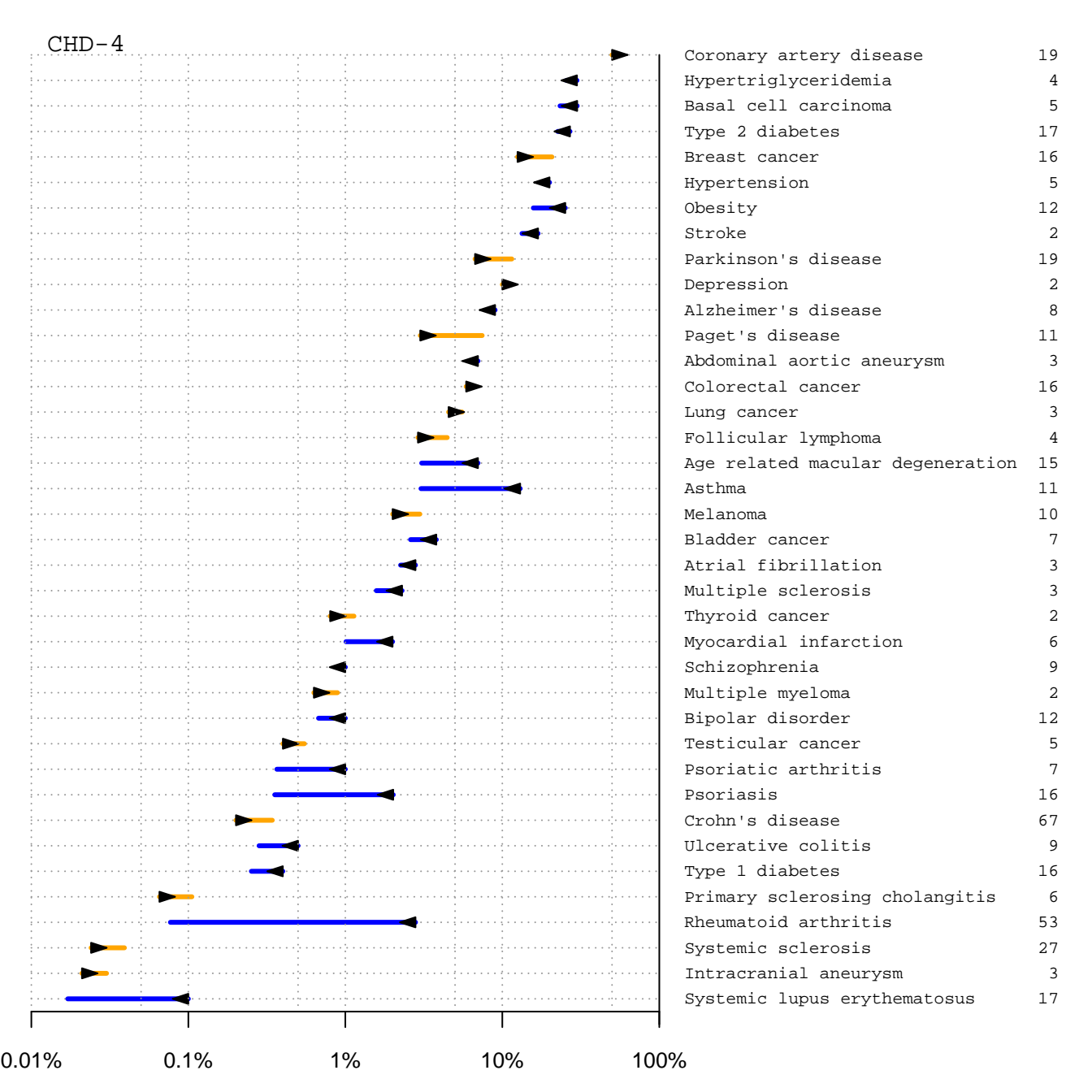

CHD-5

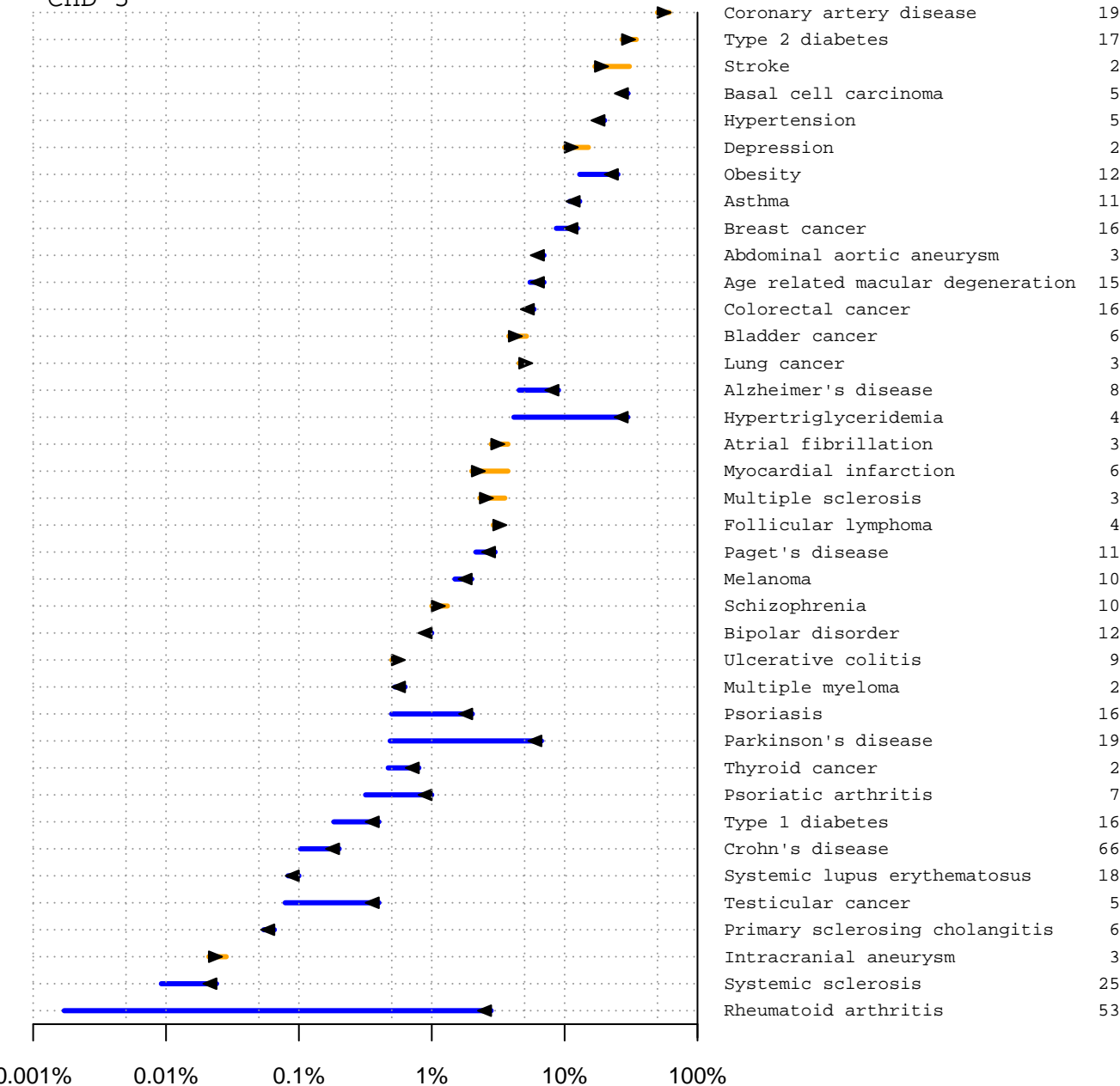

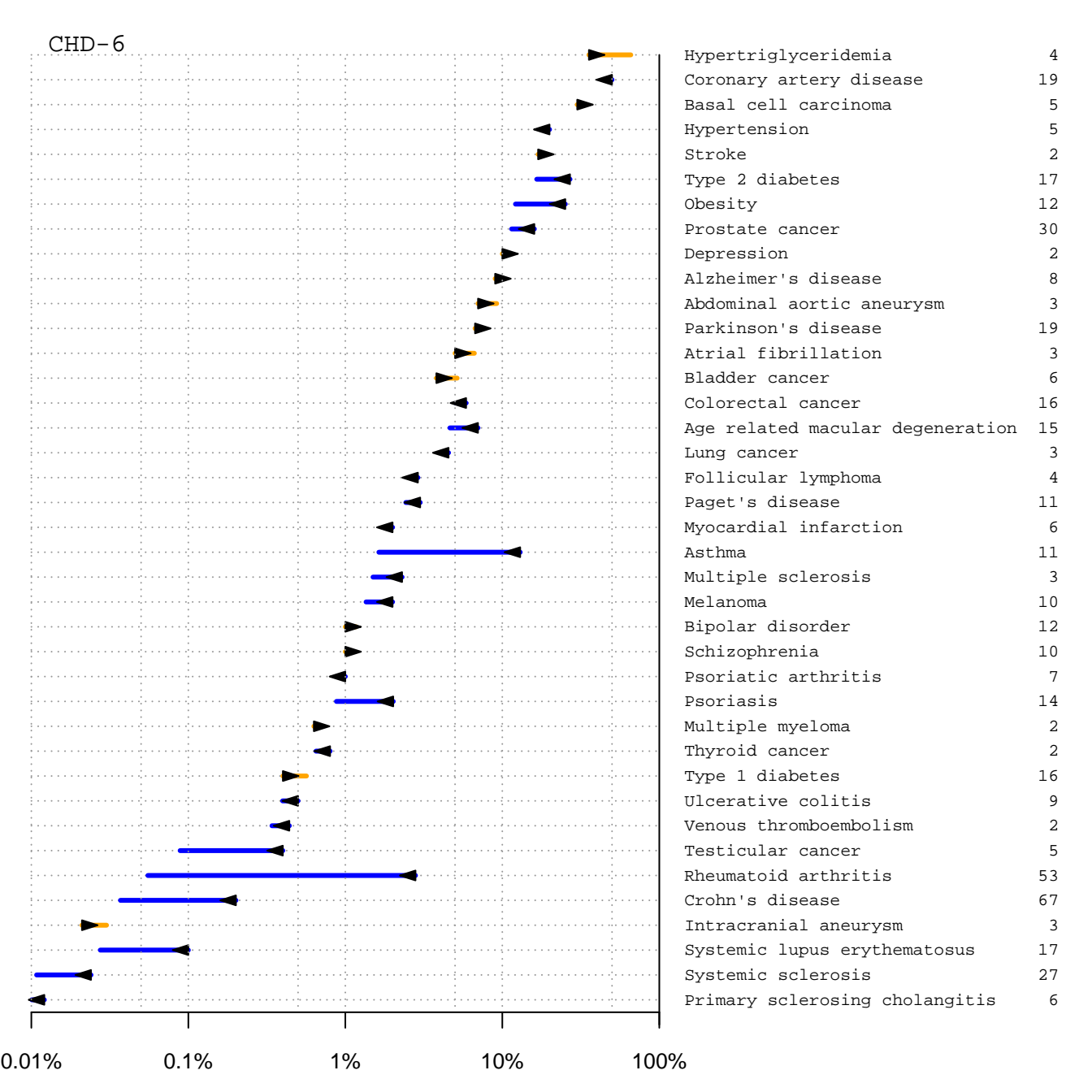

CHD-7

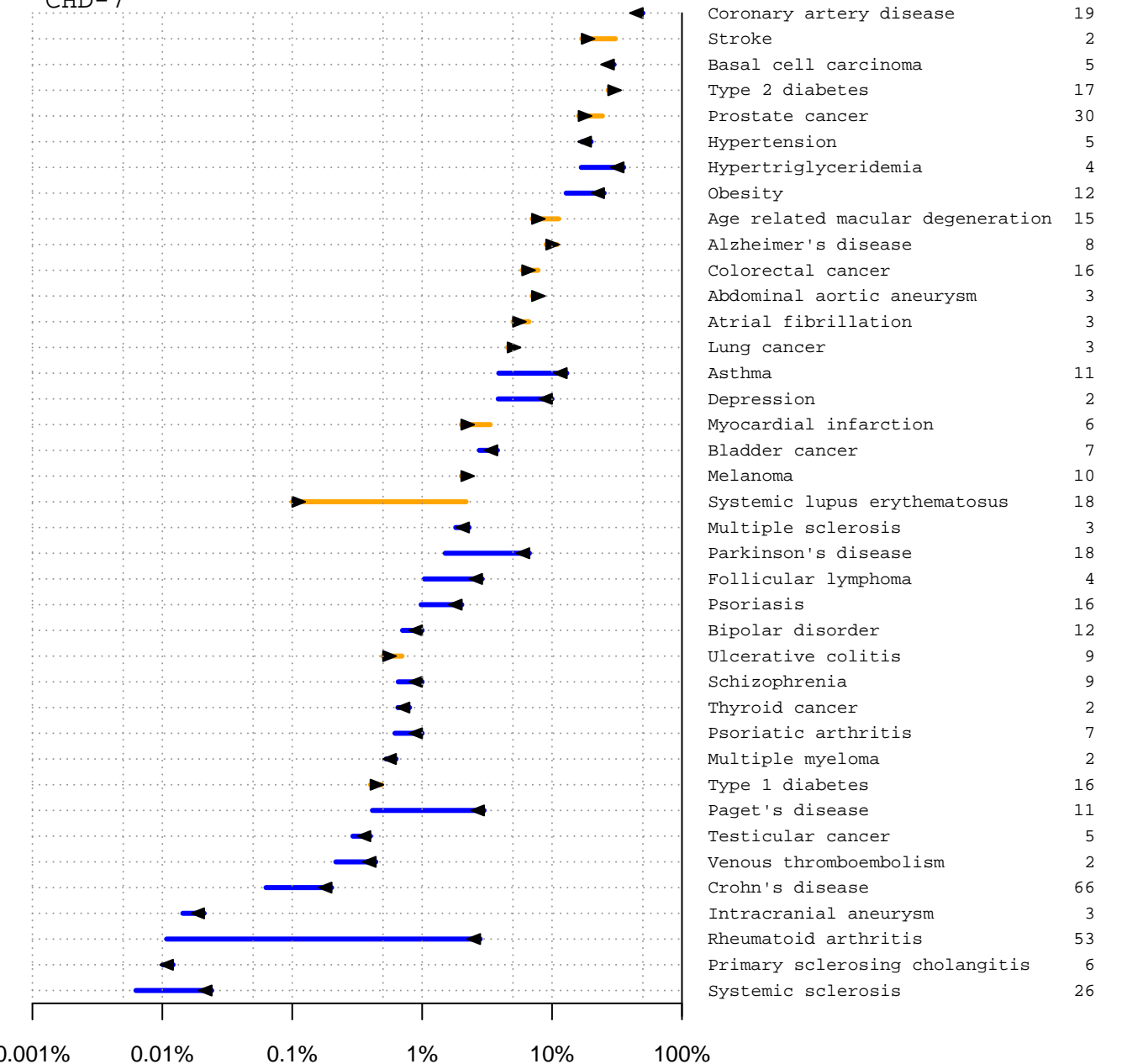

CHD-8

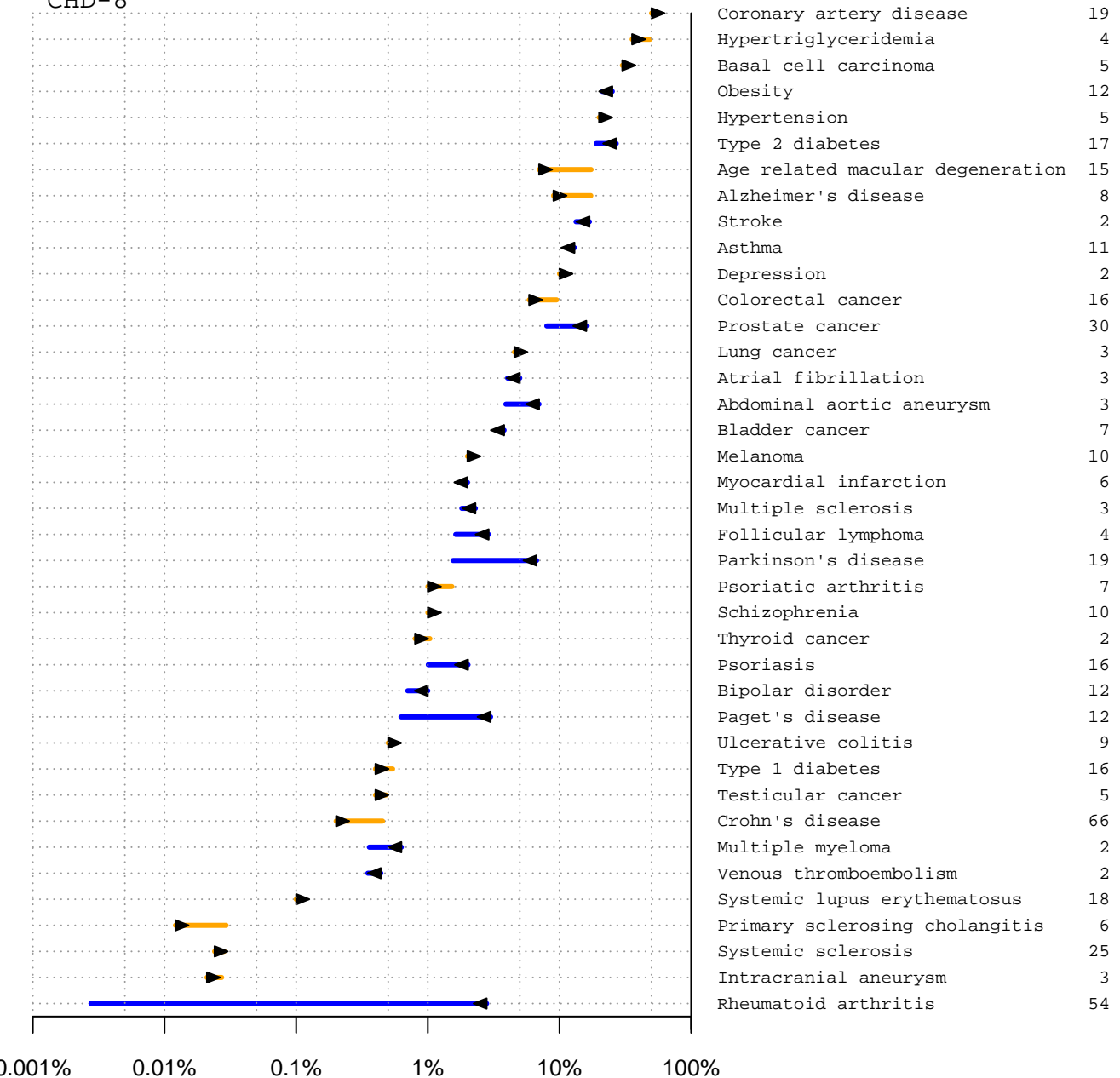

Supplement: Additional file 5 — Risk-o-grams. Figure showing the risk-o-gram plots depicting the genotypic risk for all eight subjects. [file gm462-S5.PDF]
