# Supplementary material for: Whole genome sequencing in support of wellness and health maintenance
Source: Genome Med. 2013 Jun 27;5(6):58. doi: 10.1186/gm462 (PMC3967117; doi:10.1186/gm462)
Supplement: Additional file 4 — Disease categories. Table showing the classification of clinical phenotypes into various disease categories for clinical risk assessment [file gm462-S4.PDF]

Additional file 4: Clinical attributes included in risk categories

Immunological (**IMM**)	Basophil counts<br>White blood cell count<br>Self-reported infection frequency	Eosinophil count<br>Self-reported allergies
Metabolic (**MET**)	Physical summary score<br>% Body Fat<br>Total cholesterol<br>HDL-cholesterol<br>Fasting blood glucose	Body Mass Index<br>Waist to Hip ratio<br>Triglycerides<br>LDL cholesterol<br>Fasting insulin
Cardiovascular (**CVD**)	Systolic blood pressure<br>Resting Heart Rate<br>Sub-Endocardial Viability Ratio<br>Hyperemia (mean)<br>Augmentation index adjusted to 75 bpm heart rate	Diastolic blood pressure<br>Neutrophil:Lymphocyte ratio<br>Flow-Mediated vaso-Dilation<br>Pulse Wave Velocity
Musculo-Skeletal (**MSK**)	Serum calcium<br>Vitamin b12<br>Bone Mineral Density	Bioavailable testosterone<br>Vitamin d25<br>Bodily Pain Score
Respiratory (**RES**)	VO2 maximum<br>Physical function score	Shortness of breath score
Cognitive (**COG**)	Epworth Sleep scale<br>Short-term memory	ESSI Enriched social support score<br>Executive Function
Psychiatric (**PSY**)	Beck Depression Index<br>Alcohol use (drinks/week)	Mental Health Summary score<br>GAD7 generalized anxiety measure
Oncology (**ONC**)	Any history of cancer<br>*PSA*	Estradiol

All scores were normalized to z-scores across the entire CHDWB cohort, and averaged over the first three visits to obtain relative values that can be combined or averaged as described in the methods. Details may be obtained from the corresponding author at Georgia Tech (GG).
