# Supplementary material for: Whole genome sequencing in support of wellness and health maintenance
Source: Genome Med. 2013 Jun 27;5(6):58. doi: 10.1186/gm462 (PMC3967117; doi:10.1186/gm462)
Supplement: Additional file 6 — Summary clinical profile. This one-page summary of the joint genomic and clinical profile for a hypothetical individual suggests how health professionals might present data to patients. The radar plot at the top summarizes health risks for one or more diseases of interest in each of the health domains shown in Figure 3, with the outer ring representing very high genotypic risk and the inner ring very low risk. The size of each point shows the magnitude of clinical risk in the same domain, with green dots highlighting concordant high risk, red dots discordant low genetic and high clinical risk, and blue dots discordant high genetic but low clinical risk. These are shown in more detail below, where the frequency distribution summarizes the genetic risk estimates across a relevant comparison population, and the box-and-whisker plots show the first two standard deviation intervals either side of the mean for associated clinical parameters. Colored points indicate the position of the individual relative to the comparison population. For example, this individual has relatively high genetic risk of depression, which corresponds to high Beck Depression Index, low mental health summary score, and very low social function (possibly suggesting an area for behavioral modification). In the cardiovascular domain, she has very high blood pressure despite low genetic risk of hypertension, and this contributes to relatively high Framingham Risk Score for cardiovascular disease (CVD risk) despite normal arterial stiffness. In the metabolic domain, the data show that she is currently healthy, but a high genetic risk suggests a need for ongoing surveillance. Finally, the report would mention rare variants of various types, including homozygous deleterious alleles that are known to promote rare conditions, or to be protective, as well as carrier status for rare variants that might be of interest in the context of family planning. In addition to this summary report, we envision that a mo [file gm462-S6.PDF]

Additional File 6 Genomic and Clinical Risk Summary

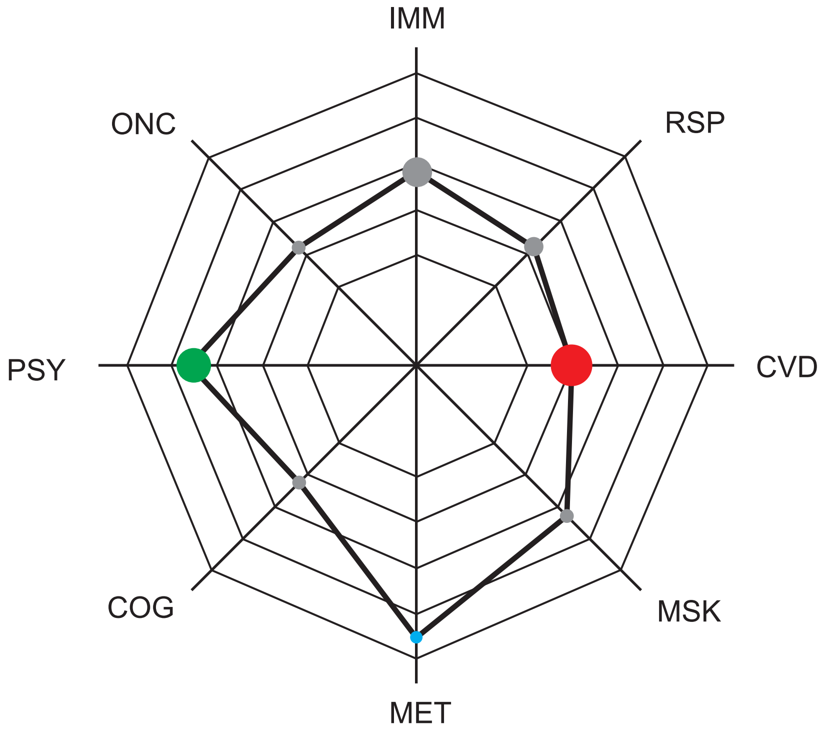

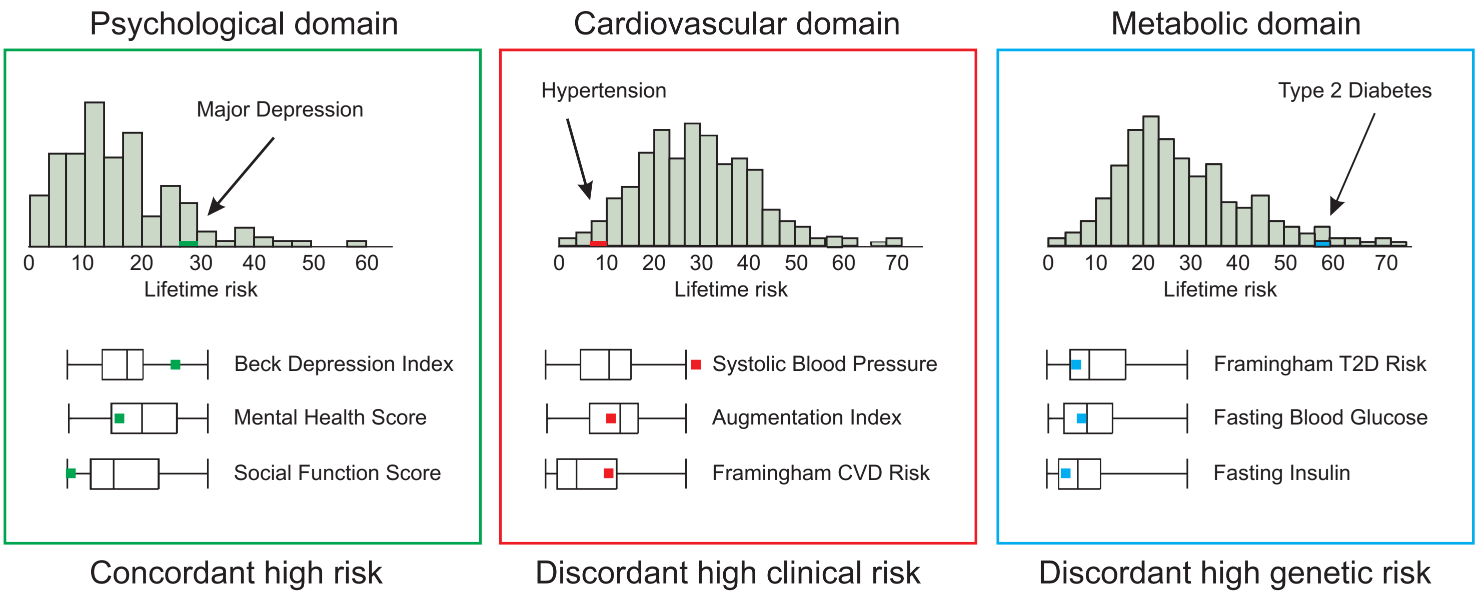

Homozygous Variants of Interest

rs28940292	c.2219G>C	p.W740S	MFN2	Thought to cause Charcot-Marie-Tooth disease
rs2266780	c.923A>G	p.E308G	FMO3	Cause of mild trimethylaminurea (Fish-Odor Syndrome)
rs7412	c.526C>T	p.R176C	APOE2	On protective allele for Alzheimer’s Disease, but may promote weight gain

Heterozygous Carrier Status

F508del-CFTR	CFTR	Known causal variant for cystic fibrosis
15q12 CNV	chr15:21490300..25698400	Duplication in ubiquitin ligase UBE3A associated with autism
